# Liver injury in hospitalized patients with COVID-19: An International observational cohort study

**DOI:** 10.1371/journal.pone.0277859

**Published:** 2023-09-13

**Authors:** Bharath Kumar Tirupakuzhi Vijayaraghavan, Saptarshi Bishnu, Joaquin Baruch, Barbara Wanjiru Citarella, Christiana Kartsonaki, Aronrag Meeyai, Zubair Mohamed, Shinichiro Ohshimo, Benjamin Lefèvre, Abdulrahman Al-Fares, Jose A. Calvache, Fabio Silvio Taccone, Piero Olliaro, Laura Merson, Neill K.J. Adhikari, the ISARIC Clinical Characterisation Group

**Affiliations:** 1 Department of Critical Care Medicine, Apollo Main Hospital, Chennai, India and Honorary Senior Fellow, The George Institute for Global Health, New Delhi, India; 2 ISARIC, Pandemic Science Institute, University of Oxford, Oxford, United Kingdom; 3 Department of Hepatology, Apollo Main Hospital, Chennai, India and Department of Gastroenterology and Hepatology, Sharanya Multi-specialty Hospital, Burdwan, India; 4 MRC Population Health Research Unit, Clinical Trials Service Unit and Epidemiological Studies Unit, Nuffield Department of Population Health, University of Oxford, Oxford, United Kingdom; 5 Department of Critical Care Medicine, Amrita Institute of Medical Sciences, Kochi, Kerala, India; 6 Department of Emergency and Critical Care Medicine, Graduate School of Biomedical and Health Sciences, Hiroshima University, Hiroshima, Japan; 7 Université de Lorraine, CHRU-Nancy, Service des Maladies Infectieuses et Tropicales, Nancy, France; 8 Université de Lorraine, APEMAC, Nancy, France; 9 Department of Anaesthesia, Critical Care Medicine and Pain Medicine, Al-Amiri Hospital, Ministry of Health, Kuwait, Kuwait; 10 Kuwait Extracorporeal Life Support program, Al-Amiri Hospital, Ministry of Health, Kuwait, Kuwait; 11 Departamento de Anestesiologia, Universidad del Cauca, Popayan, Colombia; 12 Department of Anaesthesiology, Erasmus University Medical Center, Rotterdam, The Netherlands; 13 Department of Intensive Care Medicine, Erasme Hospital, Universite Libre de Bruxelles, Brussels, Belgium; 14 Interdepartmental Division of Critical Care Medicine, University of Toronto and Department of Critical Care Medicine, Sunnybrook Health Sciences Centre, Toronto, Canada; Heidelberg University Hospital, GERMANY

## Abstract

**Background:**

Using a large dataset, we evaluated prevalence and severity of alterations in liver enzymes in COVID-19 and association with patient-centred outcomes.

**Methods:**

We included hospitalized patients with confirmed or suspected SARS-CoV-2 infection from the International Severe Acute Respiratory and emerging Infection Consortium (ISARIC) database. Key exposure was baseline liver enzymes (AST, ALT, bilirubin). Patients were assigned Liver Injury Classification score based on 3 components of enzymes at admission: Normal; Stage I) Liver injury: any component between 1-3x upper limit of normal (ULN); Stage II) Severe liver injury: any component ≥3x ULN. Outcomes were hospital mortality, utilization of selected resources, complications, and durations of hospital and ICU stay. Analyses used logistic regression with associations expressed as adjusted odds ratios (OR) with 95% confidence intervals (CI).

**Results:**

Of 17,531 included patients, 46.2% (8099) and 8.2% (1430) of patients had stage 1 and 2 liver injury respectively. Compared to normal, stages 1 and 2 were associated with higher odds of mortality (OR 1.53 [1.37–1.71]; OR 2.50 [2.10–2.96]), ICU admission (OR 1.63 [1.48–1.79]; OR 1.90 [1.62–2.23]), and invasive mechanical ventilation (OR 1.43 [1.27–1.70]; OR 1.95 (1.55–2.45). Stages 1 and 2 were also associated with higher odds of developing sepsis (OR 1.38 [1.27–1.50]; OR 1.46 [1.25–1.70]), acute kidney injury (OR 1.13 [1.00–1.27]; OR 1.59 [1.32–1.91]), and acute respiratory distress syndrome (OR 1.38 [1.22–1.55]; OR 1.80 [1.49–2.17]).

**Conclusions:**

Liver enzyme abnormalities are common among COVID-19 patients and associated with worse outcomes.

## Introduction

Coronavirus Disease 2019 (COVID-19) caused by the Severe Acute Respiratory Syndrome Coronavirus 2 (SARS-CoV-2) has thus far resulted in over 6.9 million deaths globally [1] and continues to contribute to substantial morbidity and mortality. Our understanding of COVID-19 has considerably evolved from the time the first case was reported in December 2019, and while pulmonary manifestations predominate, multi-organ involvement is well-described [2].

As a component of multi-organ involvement, liver injury, defined by elevated liver enzymes (alanine aminotransferase [ALT], aspartate aminotransferase [AST], and serum bilirubin), has been reported in 15–65% of patients [3–7]. Abnormalities in liver enzymes have been associated with severe COVID-19 and an increased risk of death [4, 6, 8, 9]. Multiple mechanisms may contribute to liver injury in COVID-19, including direct viral toxicity, endothelial damage and immune dysfunction [2]. Drugs used to treat patients with COVID-19, such as remdesivir, may also contribute to liver injury [10]. However, current information on the extent and severity of liver enzyme derangements and their implications for clinical practice come predominantly from small single-centre studies.

In January 2020, the International Severe Acute Respiratory and emerging Infection Consortium (ISARIC) [11], in partnership with the World Health Organization (WHO), activated the ISARIC-WHO Clinical Characterisation Protocol and case report form (CRF) to collect data on demographics, illness severity, treatment strategies and outcomes for hospitalized patients with COVID-19 [12, 13]. ISARIC hosts data for the largest world-wide cohort of hospitalized COVID-19 patients.

Using the ISARIC dataset, we evaluated the prevalence and severity of derangements in liver enzymes among patients admitted to hospital with COVID-19 and the association between liver enzymes measured during the first 24 hours and patient-centred hospital outcomes.

## Methods

### Study design and ethics

The ISARIC-WHO Clinical Characterization Protocol for Severe Emerging Infections provided the framework for prospective observational data collection on hospitalised patients with COVID-19. The protocol, CRFs, consent forms, and study information are available online [14]. These CRFs were developed to standardise clinical data collection on patients admitted with suspected or confirmed COVID-19, with clinical data on more than 900,000 individuals hospitalised with confirmed COVID-19 infection across 64 countries stored in a central database (as of June 2022). The CRFs collect data on demographics, pre-existing comorbidities and risk factors, signs and symptoms during the acute phase, supportive care and treatments received during hospitalisation, and outcomes [14].

This observational study required no change to clinical management. The ISARIC-WHO Clinical Characterisation Protocol was approved by the World Health Organization Ethics Review Committee (RPC571 and RPC572 on 25 April 2013). Institutional approval was additionally obtained by participating sites including the South Central Oxford C Research Ethics Committee in England (Ref 13/SC/0149) and the Scotland A Research Ethics Committee (Ref 20/SS/0028) for the United Kingdom, representing the majority of the data. Other institutional and national approvals were obtained by participating sites as per local requirements. Regionally appropriate decisions regarding a waiver or requirement of patient consent were made by each committee and implemented at the sites. A statistical analysis plan was developed *a priori* for this study and reviewed by the ISARIC Clinical-Analytic Team as well as by partner sites and collaborators (S1 File). This study is being reported as per the Strengthening the Reporting of Observational Studies in Epidemiology (STROBE) guidelines [15] (S3 Table).

### Study population

We included all individuals in the ISARIC database with laboratory confirmed or suspected SARS-CoV-2 infection admitted to hospital from 30/01/2020 to 21/09/2021 for the primary analysis. For the sensitivity analysis, we included only patients with laboratory-confirmed SARS-CoV-2 infection. We excluded patients for whom information on liver enzyme tests or clinical outcomes were not available.

### Variables and definitions

Serum bilirubin, ALT, and AST measured at or within 24 hours of hospital admission were considered as the liver enzymes for this analysis, based on availability in the ISARIC database. For the purposes of this analysis, upper limits of normal (ULN) for serum bilirubin, ALT and AST were taken as 1 mg/dL, 40 U/L and 40 U/L respectively. In the absence of an established scale for liver injury based in these laboratory tests, we adapted our criteria from previous studies that have used similar approaches [8, 9, 16–18]. Each patient was assigned a Liver Injury Classification (LIC) score at baseline based on the 3 components of LFT on admission: stage 0) Normal: all 3 components ≤ULN; Stage I) Liver injury: any 1 component between 1-3x ULN; Stage II) Severe liver injury: any 1 component ≥3x ULN.

#### Main exposures and outcomes

For the primary research question, the main exposure was baseline liver enzymes (defined as above) and the primary outcome was hospital mortality. Secondary outcomes included admission to an intensive care unit (ICU); receipt of oxygen therapy, non-invasive ventilation (NIV) or invasive ventilation, inotropes/vasopressors, and renal replacement therapy; and the durations of hospital and ICU stay. We also performed additional analyses analysis examining the association between baseline liver enzymes and specific complications not present at admission and developing in hospital, including acute respiratory distress syndrome (ARDS), hemodynamic complications, acute kidney injury, sepsis, and hematological and neurological complications. Definitions for these complications are available from the ISARIC CRF completion guide [19]. Other baseline exposures were included based on biological relevance: comorbidities, age (in ten-year bands), sex, and depending on the model, ICU admission and receipt of oxygen.

### Statistical analysis

Categorical variables were summarized as counts and percentages and continuous variables as mean ± standard deviation (SD) or median (first quartile [Q3], third quartile [Q3]), depending on distribution. The prevalence of liver enzyme derangements at baseline was estimated using the LIC classification described. The cumulative probability of a patient remaining in hospital or ICU (i.e., length of stay) was plotted graphically, stratified by discharge vital status and age.

We used logistic regression to determine the association between exposure variables and outcomes, expressed as odds ratios (ORs) with 95% confidence intervals (CIs). Dichotomous outcomes were analysed using a binomial distribution and a logit link. As mentioned above, covariates were selected based on the biological relevance. For the remaining covariates, those with P <0.20 in univariable analysis were retained for the multivariable analysis, and backwards elimination was used for model selection. To account for potential effect modifications, two-way interactions were evaluated between LIC and age categories, ICU admission, and receipt of remdesivir. Missing data were not imputed, and analyses used complete cases. In a sensitivity analysis (primary outcome), we included only laboratory confirmed patients. We used R4.1.2 [R Core Team. R: A language and environment for statistical computing. R Foundation for Statistical Computing, Vienna, Austria. https://www.R-project.org/] for statistical analysis.

#### Deviations from original analysis plan

We originally planned to examine the association between receipt of remdesivir and new liver enzyme elevation, as well as at trends of liver enzymes during the hospital stay. Both these analyses were not possible due to the high degree of missingness for these variables in the dataset.

## Results

From a total of 708,052 patients in the ISARIC database as of 21^st^ September 2021, we included 17,531 patients after eliminating those with missing baseline information on liver enzyme tests (n = 686,122 patients) and outcomes (4399 patients). Baseline characteristics of included patients are presented by stages of LIC classification in Table 1. The mean age was 56.5 (SD 20.3) years, and 60.0% of the patients were male. Hypertension (33.9%) and diabetes (31.3%) were the most common comorbidities. Around 3.0% of the cohort had chronic liver disease. Cough (60.0%) and fever (59.0%) were the most common presenting symptoms of COVID-19.

**Table 1:** Baseline characteristics by stages of liver injury in the ISARIC clinical characterisation database (n = 17531).

Characteristic	Stage 0 (n = 8002)	Stage 1 (n = 8099)	Stage 2 (n = 1430)
**Age (mean** **+** **SD)**	56.0 + 21.5	60.8 + 18.8	57.4 + 19.0
**Sex (n, %)**			
Male	3732 (46.6)	2661 (32.9)	435 (30.4)
Female	4264 (53.3)	5421 (66.9)	992 (69.4)
Unknown	6 (0.1)	17 (0.2)	3 (0.2)
**Comorbidities**			
Hypertension (n, %)			
No	4524 (56.5)	4010 (49.5)	732 (51.2)
Yes	2576 (32.2)	2890 (35.7)	472 (33)
Yes	2576 (32.2)	2890 (35.7)	472 (33)
Unknown	902 (11.3)	1199 (14.8)	226 (15.8)
Chronic liver disease (n, %)			
No	7736 (96.7)	7657 (94.5)	1284 (89.8)
Yes	153 (1.9)	252 (3.1)	92 (6.4)
Unknown	113 (1.4)	190 (2.3)	54 (3.8)
Chronic kidney disease (n, %)			
No	6992 (87.4)	7141 (88.2)	1209 (84.5)
Yes	781 (9.8)	619 (7.6)	143 (10)
Unknown	229 (2.9)	339 (4.2)	78 (5.5)
Chronic neurological disorder (n, %)			
No	7198 (90)	7259 (89.6)	1252 (87.6)
Yes	561 (7)	502 (6.2)	101 (7.1)
Unknown	243 (3)	338 (4.2)	77 (5.4)
Chronic pulmonary disease (n, %)			
No	6932 (86.6)	6995 (86.4)	1217 (85.1)
Yes	858 (10.7)	794 (9.8)	137 (9.6)
Unknown	212 (2.6)	310 (3.8)	76 (5.3)
Malignant neoplasm (n, %)			
No	7301 (91.2)	7298 (90.1)	1249 (87.3)
Yes	477 (6)	491 (6.1)	115 (8)
Unknown	224 (2.8)	310 (3.8)	66 (4.6)
Obesity (n, %)			
No	5731 (71.6)	5292 (65.3)	917 (64.1)
Yes	822 (10.3)	1136 (14)	200 (14)
Unknown	1449 (18.1)	1671 (20.6)	313 (21.9)
**Symptoms**			
Cough (n, %)			
No	3354 (41.9)	2498 (30.8)	491 (34.3)
Yes	4385 (54.8)	5281 (65.2)	838 (58.6)
Unknown	263 (3.3)	320 (4.0)	101 (7.1)
History of fever (n, %)			
No	3470 (43.4)	2512 (31)	492 (34.4)
Yes	4222 (52.8)	5259 (64.9)	850 (59.4)
Unknown	310 (3.9)	328 (4.0)	88 (6.2)
lost altered sense of smell (n, %)			
No	4986 (62.3)	4668 (57.6)	830 (58)
Yes	397 (5)	446 (5.5)	63 (4.4)
Unknown	2619 (32.7)	2985 (36.9)	537 (37.6)
Shortness of breath (n, %)			
No	4096 (51.2)	2666 (32.9)	428 (29.9)
Yes	3652 (45.6)	5199 (64.2)	937 (65.5)
Unknown	254 (3.2)	234 (2.9)	65 (4.5)

45.6% of patients had normal liver enzymes, 46.2% had stage 1 liver injury, and 8.2% of the cohort had stage 2 liver injury (Table 1). Patients met criteria for liver injury predominantly through elevations in AST or ALT (S2 Table).

Treatments received during hospital stay are presented by stages of LIC in Table 2. Admission to an ICU for patients with LIC stages 0, 1, and 2 occurred in 1514 (18.9%), 2834 (35.0%) and 573 (40.1%) patients, respectively. Oxygen supplementation was provided in 3838 (48.0%), 5692 (70.3%), and 1086 (75.9%) of patients. 782 patients (9.8%) in stage 0, 1722 (21.3%) in stage 1, and 386 (27.0%) in stage 2 received invasive ventilation. Corticosteroids were administered to 2294 (28.7%, stage 0), 3446 (42.5%, stage 1), and 656 (45.9%, stage 2) patients. The median (IQR) length of hospital stay for stages 0, 1 and 2 were 9 (5–15) days, 8 (5–16) days, and 9 (4–14) days, respectively. Similarly, the median (IQR) length of ICU stay for stages 0, 1 and 2 were 7 (4–15) days, 8 (5–17) days, and 9 (3–14) days, respectively (S1 and S2 Figs).

**Table 2:** Interventions and treatments during hospital stay among patients in the ISARIC clinical characterisation database with different levels of liver injury (n = 17531).

Intervention	Stage 0 (n = 8002)	Stage 1 (n = 8099)	Stage 2 (n = 1430)
ICU admission (n, %)			
No	6375 (79.7)	5139 (63.5)	823 (57.6)
Yes	1514 (18.9)	2834 (35.0)	573 (40.1)
Unknown	113 (1.4)	126 (1.6)	34 (2.4)
Oxygen therapy (n, %)			
No	4123 (51.5)	2377 (29.3)	338 (23.6)
Yes	3838 (48.0)	5692 (70.3)	1086 (75.9)
Unknown	41 (0.5)	30 (0.4)	6 (0.4)
Non-invasive ventilation (n, %)			
No	7136 (89.2)	6494 (80.2)	1149 (80.3)
Yes	712 (8.9)	1444 (17.8)	260 (18.2)
Unknown	154 (1.9)	161 (2.0)	21 (1.5)
Invasive ventilation (n, %)			
No	7141 (89.2)	6308 (77.9)	1034 (72.3)
Yes	782 (9.8)	1722 (21.3)	386 (27)
Unknown	79 (1.0)	69 (0.9)	10 (0.7)
Inotropes/vasopressors (n, %)			
No	7088 (88.6)	6381 (78.8)	1064 (74.4)
Yes	701 (8.8)	1466 (18.1)	322 (22.5)
Unknown	213 (2.7)	252 (3.1)	44 (3.1)
Corticosteroids (n, %)			
No	5452 (68.1)	4366 (53.9)	703 (49.2)
Yes	2294 (28.7)	3446 (42.5)	656 (45.9)
Unknown	256 (3.2)	287 (3.5)	71 (5.0)
Antiviral (n, %)			
No	4250 (53.1)	4897 (60.5)	964 (67.4)
Yes	1530 (19.1)	1985 (24.5)	303 (21.2)
Unknown	2222 (27.8)	1217 (15)	163 (11.4)
Antibiotics (n, %)			
No	1407 (17.6)	1038 (12.8)	177 (12.4)
Yes	4163 (52)	5728 (70.7)	1079 (75.5)
Unknown	2432 (30.4)	1333 (16.5)	174 (12.2)

The crude risk of death was 14.3% in stage 0, 23.4% in stage 1, and 32.7% in stage 2 (Table 3).

**Table 3:** Unadjusted risk of death by LIC score in the ISARIC clinical characterisation data base (n = 17531).

Outcome	Stage 0 (n = 8002)	Stage 1 (n = 8099)	Stage 2 (n = 1430)
Death (n, %)	1145 (14.3)	1893 (23.4)	467 (32.7)
Discharge (n, %)	6857 (85.7)	6206 (76.6)	963 (67.3)

In multivariable analysis (Table 4), compared to normal, stages 1 and 2 were associated with higher odds of mortality (OR 1.53 [1.37–1.71]; OR 2.50 [2.10–2.96]), ICU admission (OR 1.63 [1.48–1.79]; OR 1.90 [1.62–2.23]) and invasive mechanical ventilation (OR 1.43 [1.27–1.70]; OR 1.95 [1.55–2.45]).

**Table 4:** Multivariable analysis for the association between LIC score and different outcomes (death, ICU admission, and IMV) among patients in the ISARIC clinical characterisation database (n = 17531).

A. Odds ratio for the association between death and LIC score, adjusted for comorbidities, symptoms, and demographics.			
Term	Odds Ratio	95% CI	P-value
LIC 0	Ref	ref	ref
LIC 1	1.53	(1.37–1.71)	<0.01
LIC 2	2.50	(2.1–2.96)	<0.01
B. Odds ratio for the association between ICU admission and LIC score, adjusted for comorbidities, symptoms, and demographics.			
Term	Odds Ratio	95% CI	P-value
LIC 0	Ref	ref	ref
LIC 1	1.63	(1.48–1.79)	<0.01
LIC 2	1.90	(1.62–2.23)	<0.01
C. Odds ratio for the association between IMV treatment and LIC score, adjusted for comorbidities, symptoms, and demographics.			
Term	Odds Ratio	95% CI	P-value
LIC 0	Ref	ref	ref
LIC 1	1.43	(1.27–1.70)	<0.01
LIC 2	1.95	(1.55–2.45)	<0.01

*The model was adjusted for: hematologic disease, chronic kidney disease, chronic neurological disorder, chronic pulmonary disease, dementia, diabetes, hypertension, liver disease, malignant neoplasm, obesity, smoking, age group, sex, cough, headache, shortness of breath, vomiting/nausea, and ICU admission.

*The model was adjusted for: AIDS/HIV, cardiac disease, pulmonary disease, asthma, chronic kidney disease, chronic neurological disorder, dementia, diabetes, hypertension, liver disease, obesity, malignant neoplasm, rheumatologic disorder, smoking, age group, sex, history of fever, shortness of breath.

*The model was adjusted for: AIDS/HIV, chronic cardiac disease, chronic hematologic disease, chronic kidney disease, chronic neurological disorder, chronic pulmonary disease, diabetes, hypertension, liver disease, malignant neoplasm, obesity, rheumatologic disorder, smoking, age group, sex, shortness of breath, vomiting nausea, ICU admission

Associations of LIC with complications are shown in Table 5. When comparing to stage 0, stage 1 and 2 of LIC were associated with a higher odds of developing sepsis (OR 1.38 [1.27–1.50]; OR 1.46 [1.25–1.70]), acute kidney injury (OR 1.13 [1.00–1.27]; OR 1.59 [1.32–1.91]), and ARDS (OR 1.38 [1.22–1.55]; OR 1.80 [1.49–2.17]). When comparing to stage 0, only stage 2 was associated with higher odds of hemodynamic (OR 1.46 [1.20–1.77]) and neurological (OR 1.58 [1.08–2.27]) complications.

**Table 5:** Multivariable analysis for the association between in-hospital complications and liver injury score, while adjusted for demographics, comorbidities, treatments, and symptoms. Reference category is stage 0 (n = 17531).

Complication	Stage 0 (n = 8002)	Stage 1 (n = 8099)	Stage 2 (n = 1430)	OR (95% CI) for Stage 1	OR (95% CI) for Stage 2
Sepsis					
No	4351 (54.4)	3108 (38.4)	495 (34.6)	1.38 (1.27–1.5)	1.46 (1.25–1.7)
Yes	3274 (40.9)	4550 (56.2)	843 (59)		
Unknown	377 (4.7)	441 (5.4)	92 (6.4)		
ARDS					
No	6445 (80.5)	5531 (68.3)	896 (62.7)	1.38 (1.22–1.55)	1.8 (1.49–2.17)
Yes	1003 (12.5)	1864 (23)	395 (27.6)		
Neurological					
No	7423 (92.8)	7405 (91.4)	1281 (89.6)	0.95 (0.74–1.24)	1.58 (1.08–2.27)
Yes	144 (1.8)	171 (2.1)	48 (3.4)		
Unknown	435 (5.4)	523 (6.5)	101 (7.1)		
Pulmonary					
No	6324 (79)	6120 (75.6)	1069 (74.8)	0.94 (0.79–1.11)	1.13 (0.87–1.47)
Yes	302 (3.8)	399 (4.9)	90 (6.3)		
Unknown	1376 (17.2)	1580 (19.5)	271 (19)		
Hematological					
No	6563 (82)	6362 (78.6)	1059 (74.1)	0.85 (0.76–0.96)	1.06 (0.88–1.27)
Yes	995 (12.4)	1193 (14.7)	270 (18.9)		
Unknown	444 (5.5)	544 (6.7)	101 (7.1)		
Hemodynamic					
No	6788 (84.8)	6427 (79.4)	1059 (74.1)	1.11 (0.98–1.26)	1.46 (1.2–1.77)
Yes	779 (9.7)	1166 (14.4)	274 (19.2)		
Unknown	435 (5.4)	506 (6.2)	97 (6.8)		
Acute kidney injury					
No	6630 (82.9)	6211 (76.7)	999 (69.9)	1.13 (1.00–1.27)	1.59 (1.32–1.91)
Yes	893 (11.2)	1271 (15.7)	311 (21.7)		
Unknown	479 (6)	617 (7.6)	120 (8.4)		

Analyses adjusted for comorbidities, treatments, symptoms, and demographics.

**S1 Table** provides results of the sensitivity analysis that included only patients with lab-confirmed COVID-19. These results were largely consistent with the primary analysis.

## Discussion

Our analysis demonstrates that abnormalities in liver enzymes are common at admission in patients hospitalized with COVID-19. In our study, increasing severity of liver injury, as defined by abnormalities of transaminases or bilirubin, was associated with higher odds of mortality, ICU admission, and mechanical ventilation. Stage 1 and 2 were also associated with a higher odds of developing complications such as sepsis, AKI, and ARDS.

Previous smaller studies of patients with COVID-19 have shown similar results. Early data (n = 482) from China [20] found that nearly 30% of patients demonstrated liver enzyme abnormalities at baseline, with a higher unadjusted risk of mortality. In another small cohort (n = 147) from Germany [9], over 50% of patients had liver injury at baseline which was independently associated with mortality. In a larger cohort (n = 5771) of hospitalized patients from Hubei province [21], increasing values of AST, ALT, alkaline phosphatase, and bilirubin were associated with mortality, with AST the most deranged liver enzyme at admission for patients with severe COVID-19, and remaining high throughout hospitalization.

Results of our analysis are largely consistent with these prior studies and strengthen the evidence base with a much larger dataset. Multiple mechanisms may explain the frequency and severity of liver involvement in COVID-19. The ubiquitous distribution of the viral entry receptor, ACE2, in human tissues might imply a role for direct cytopathic effects (22). Both microvesicular and macrovesicular steatosis has been demonstrated in autopsies of patients, with SARS-CoV-2 as the only risk factor for liver injury [22]. An additional component of hypoxic hepatitis in patients with severe hypoxic respiratory failure may also contribute [23]. Additional mechanisms include hepatic vascular thrombosis, widespread systemic inflammation, and drug-related toxicity [24, 25].

Our study has several important strengths: the large size of the cohort, the availability of data from multiple countries and sites, improving generalizability of findings, a pre-specified analysis plan, and adjustment for known confounders. Limitations of the analysis include the possibility of residual confounding, the inability to evaluate trends in liver enzymes during the course of hospital stay, and the impact of antivirals. In addition, bilirubin and transaminases do not assess similar aspects of liver injury, raising the possibility that our definition of liver injury was mis-specified. Selection bias is possible, since were able to include only a fraction of the patients represented in the ISARIC database due to missing data on liver enzyme tests and outcomes. Liver enzymes are more likely to be measured in patients with signs or history of liver disease, and in patients presenting with more severe illness at presentation, indicating that the frequency of liver failure reported here is an overestimate of the hospitalised population. Also, we were unable to evaluate the association between remdesivir and other antivirals on the development of liver injury due to extensive missing data.

## Conclusion

Liver enzyme abnormalities are common among COVID-19 patients and associated with worse outcomes. Multiple mechanisms may explain the extent and severity of liver injury in COVID-19. Future research should focus on understanding these mechanisms, the impact of changes over time, and whether antivirals improve or worsen liver injury.

## Supporting information

S1 Table Sensitivity analysis excluding non-PCR-confirmed SARS-CoV-2 patients. Multivariable analysis for the association between LIC score and different outcomes (death, ICU admission, and IMV) among patients in the ISARIC clinical characterisation database (n = 17531). *The model was adjusted for: hematologic disease, chronic kidney disease, chronic neurological disorder, chronic pulmonary disease, dementia, diabetes, hypertension, liver disease, malignant neoplasm, obesity, smoking, age group, sex, cough, headache, shortness of breath, vomiting/nausea, and ICU admission.
(DOCX)

S2 Table ALT, AST, and Bilirubin relationship among patients in the ISARIC clinical characterisation database. Normal upper limits (ULN) were taken as 1 mg/dL, 40 U/L, and 40 U/L for bilirubin, ALT, and AST, respectively.
(DOCX)

S3 Table STROBE checklist: This is the checklist for reporting observational studies. (DOCX)

S1 Fig Hospital Length of stay for the different stages of liver injury stratified by age and outcome. (TIF)

S2 Fig ICU length of stay for the different stages of liver injury stratified by age and outcome. (TIF)

S1 File ISARIC collaborators: This is the full list of collaborators along with their affiliations. (XLSX)

S2 File Statistical analysis plan: This is the prespecified and original statistical analysis plan for our submission. (DOCX)
