# Supplementary material for: Liver injury in hospitalized patients with COVID-19: An International observational cohort study
Source: PLoS One. 2023 Sep 13;18(9):e0277859. doi: 10.1371/journal.pone.0277859 (PMC10499210; doi:10.1371/journal.pone.0277859)
Supplement: S1 Table — Multivariable analysis for the association between LIC score and different outcomes (death, ICU admission, and IMV) among patients in the ISARIC clinical characterisation database (n = 17531). *The model was adjusted for: hematologic disease, chronic kidney disease, chronic neurological disorder, chronic pulmonary disease, dementia, diabetes, hypertension, liver disease, malignant neoplasm, obesity, smoking, age group, sex, cough, headache, shortness of breath, vomiting/nausea, and ICU admission. (DOCX) [file pone.0277859.s001.docx]

**Supplementary Table 1**. Sensitivity analysis excluding non-PCR-confirmed SARS-CoV-2 patients. Multivariable analysis for the association between LIC score and different outcomes (death, ICU admission, and IMV) among patients in the ISARIC clinical characterisation database (n = 17531).

A. Odds ratio for the association between death and LIC score, adjusted for comorbidities, symptoms, and demographics.

| Term | Odds Ratio | 95% CI | P-value |
| --- | --- | --- | --- |
| LIC 0 | ref | ref | ref |
| LIC 1 | 1.57 | (1.36 - 1.82) | <0.01 |
| LIC 2 | 2.79 | (2.23 - 3.5) | <0.01 |

*The model was adjusted for: hematologic disease, chronic kidney disease, chronic neurological disorder, chronic pulmonary disease, dementia, diabetes, hypertension, liver disease, malignant neoplasm, obesity, smoking, age group, sex ,cough, headache, shortness of breath, vomiting/nausea, and ICU admission.

B. Odds ratio for the association between ICU admission and LIC score, adjusted for comorbidities, symptoms, and demographics.

| Term | Odds Ratio | 95% CI | P-value |
| --- | --- | --- | --- |
| LIC 0 | ref | ref | ref |
| LIC 1 | 1.80 | (1.58 - 2.04) | <0.01 |
| LIC 2 | 2.01 | (1.62 - 2.50) | <0.01 |

*The model was adjusted for: AIDS/HIV, cardiac disease, pulmonary disease, asthma, chronic kidney disease, chronic neurological disorder, dementia, diabetes, hypertension, liver disease, obesity, malignant neoplasm, rheumatologic disorder, smoking, age group, sex, history of fever, shortness of breath.

C. Odds ratio for the association between IMV treatment and LIC score, adjusted for comorbidities, symptoms, and demographics.

| Term | Odds Ratio | 95% CI | P-value |
| --- | --- | --- | --- |
| LIC 0 | ref | ref | ref |
| LIC 1 | 1.66 | (1.36 - 2.01) | <0.01 |
| LIC 2 | 2.49 | (1.82 - 3.42) | <0.01 |

*The model was adjusted for: AIDS/HIV, chronic cardiac disease, chronic hematologic disease, chronic kidney disease, chronic neurological disorder, chronic pulmonary disease, diabetes, hypertension, liver disease, malignant neoplasm, obesity, rheumatologic disorder, smoking, age group, sex, shortness of breath, vomiting nausea, ICU admission
