# Supplementary material for: Liver injury in hospitalized patients with COVID-19: An International observational cohort study
Source: PLoS One. 2023 Sep 13;18(9):e0277859. doi: 10.1371/journal.pone.0277859 (PMC10499210; doi:10.1371/journal.pone.0277859)
Supplement: S2 Table — Normal upper limits (ULN) were taken as 1 mg/dL, 40 U/L, and 40 U/L for bilirubin, ALT, and AST, respectively. (DOCX) [file pone.0277859.s002.docx]

**Supplementary Table 2**: ALT, AST, and Bilirubin relationship among patients in the ISARIC clinical characterisation database. Normal upper limits (ULN) were taken as 1 mg/dL, 40 U/L, and 40 U/L for bilirubin, ALT, and AST, respectively.

| Bilirubin | ALT | AST | | |
| --- | --- | --- | --- | --- |
|  |  | <1 ULN | 1-3 ULN | >3 ULN |
| <1 ULN |  |  |  |  |
|  | <1 ULN | 8002 | 2518 | 113 |
|  | 1-3 ULN | 1144 | 3236 | 416 |
|  | >3 ULN | 19 | 261 | 298 |
| 1-3 ULN |  |  |  |  |
|  | <1 ULN | 529 | 254 | 27 |
|  | 1-3 ULN | 85 | 333 | 79 |
|  | >3 ULN | 4 | 35 | 65 |
| >3 ULN |  |  |  |  |
|  | <1 ULN | 21 | 18 | 3 |
|  | 1-3 ULN | 1 | 25 | 15 |
|  | >3 ULN | 2 | 2 | 26 |
