# Supplementary figures and images for: Liver injury in hospitalized patients with COVID-19: An International observational cohort study
Source: PLoS One. 2023 Sep 13;18(9):e0277859. doi: 10.1371/journal.pone.0277859 (PMC10499210; doi:10.1371/journal.pone.0277859)

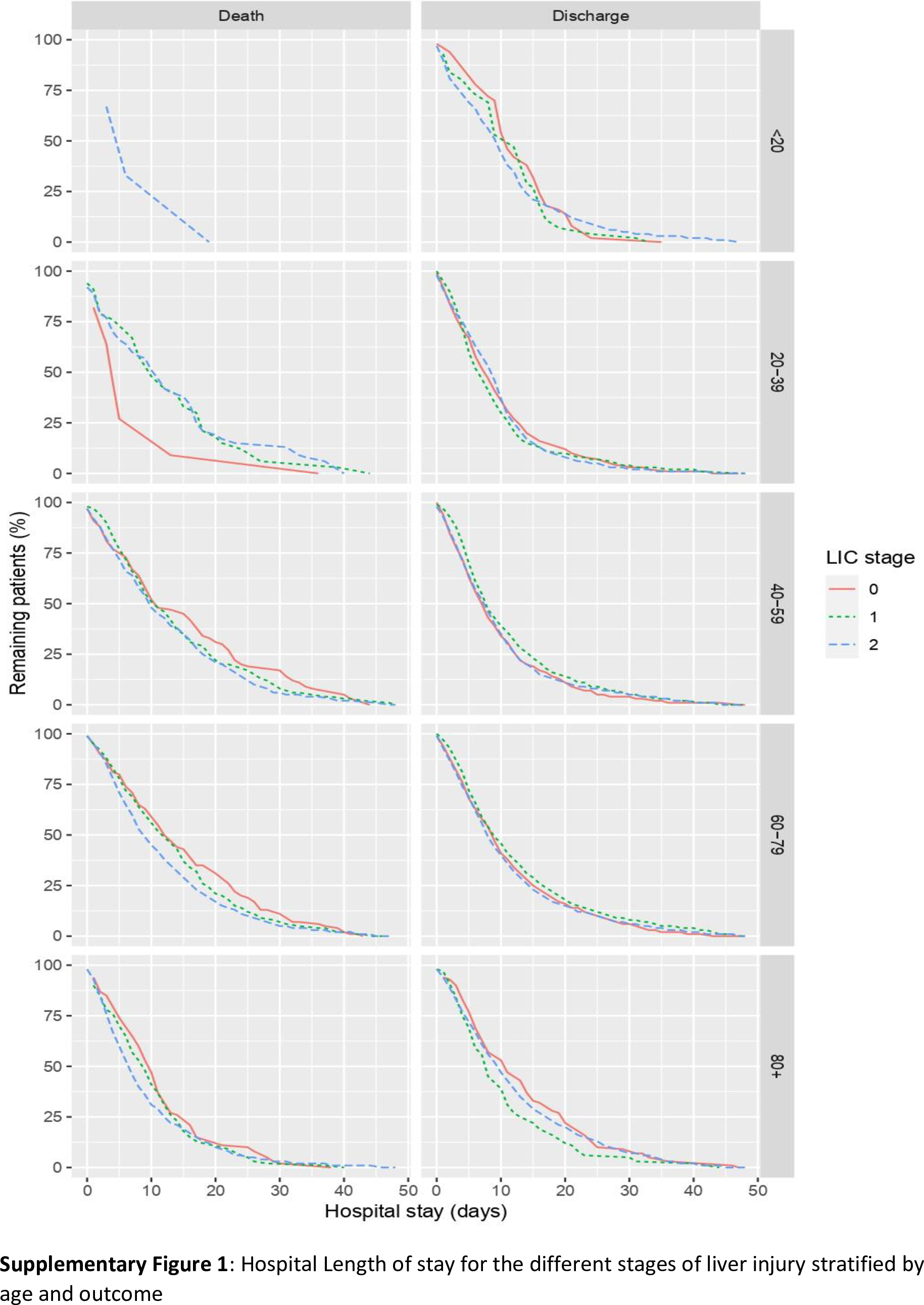

Supplement: S1 Fig — (TIF) [file pone.0277859.s004.tif]

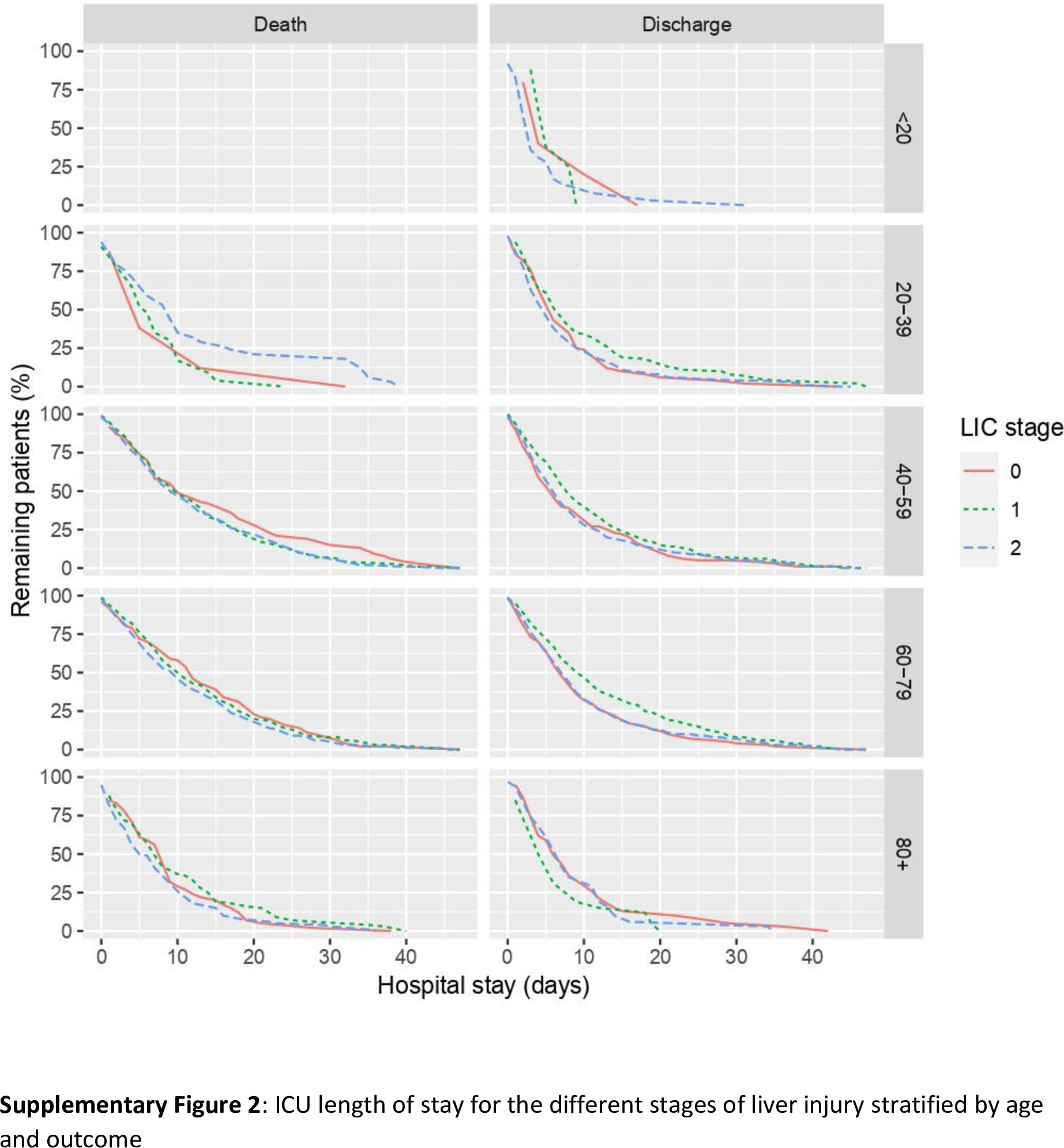

Supplement: S2 Fig — (TIF) [file pone.0277859.s005.tif]
