# Supplementary material for: Liver injury in hospitalized patients with COVID-19: An International observational cohort study
Source: PLoS One. 2023 Sep 13;18(9):e0277859. doi: 10.1371/journal.pone.0277859 (PMC10499210; doi:10.1371/journal.pone.0277859)
Supplement: S2 File — (DOCX) [file pone.0277859.s007.docx]

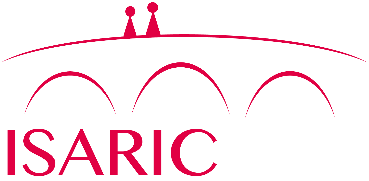


ISARIC (International Severe Acute Respiratory and Emerging Infections Consortium)

*A global federation of clinical research networks, providing a proficient, coordinated, and agile research response to outbreak-prone infectious disease*

**Analysis Plan for ISARIC International COVID-19 Patients**

*Please complete the following sections:*

| **Title of proposed research** |
| --- |
| Liver function abnormalities in patients admitted with COVID-19 and association with outcomes: A draft analysis plan using data collected by the ISARIC Collaborators |
| **Version: (Date: Day/Month/Year)** |
| **Version: 1.3**  **6^th^ July 2021** |
| **Working Group Chair (name, ORCID ID, email, institution, country)** |
| **Saptarshi Bishnu, Department of Hepatology, Apollo Hospitals, Chennai, India**  **Email:** [**saptarshibishnu@gmail.com**](about:blank)  **ORCID: 0000-0002-9001-9463** |
| **^[[1]](#footnote-1)^Working group co-chairs (name, ORCID ID, email, institution, country)** |
| **Bharath Kumar Tirupakuzhi Vijayaraghavan**  **Department of Critical Care Medicine, Apollo Hospitals, Chennai, India**  **Email:** [**bharath@icuconsultants.com**](about:blank)  **ORCID: 0000-0002-1801-0667**  **Neill KJ Adhikari**  **Interdepartmental Division of Critical Care Medicine, University of Toronto, Canada**  **Email:** [**neill.adhikari@sunnybrook.ca**](about:blank)  **ORCID: 0000-0003-4038-5382** |
| **Statistician (name, ORCID ID, email, institution, country)** |
| Aronrag Meeyai <aronrag.meeyai@ndm.ox.ac.uk>  Joaquin Baruch, 0000-0002-0806-3183, [joaquinbaruch2@gmail.com](about:blank), ISARIC |

# Introduction

This document details the initial analysis plan for publication on a subset of COVID-19 patients in the global cohort in the ISARIC database, as of 01 February 2021. There are currently 54 countries (as of 01 February 2021) contributing data and these have so far contributed data on 340,312 patients.

Derangements in liver functions have been reported in multiple small single-centre studies since the outbreak of SARS-CoV-2; elevated levels of the components of Liver Function Tests (LFT) (serum alanine aminotransferase (ALT), aspartate aminotransferase (AST), and serum bilirubin) have been reported in 15-65% of patients in these studies (1-4). LFT abnormalities were seen to be more prevalent in patients with severe COVID-19 and associated with mortality (2,4,6,7). It has also been observed that worsening of initially normal LFT parameters during the course of hospitalization was more common in patients with severe COVID-19 (8). Thus, both static and dynamic trends of LFT derangement may be associated with clinical severity and outcomes. As highlighted, current information predominantly comes from small single-centre datasets and are as such limited by design, sample-size and residual confounding.

# Participatory Approach

All contributors to the ISARIC database are invited to participate in this analysis through review and input on the statistical analysis plan and resulting publication. The outputs of this work will be disseminated as widely as possible to inform patient care and public health policy; this will include submission for publication in an international, peer-reviewed journal. ISARIC aims to include the names of all those who contribute data in the cited authorship of this publication, subject to the submission of contact details and confirmation of acceptance of the final manuscript within the required timelines, per ICMJE policies and the ISARIC publication policy.

# Research Plan

| **Summary of Research Objectives** |
| --- |
| 1. To evaluate the prevalence and severity of derangements in liver function among patients admitted with COVID-19 2. To evaluate the association between admission liver function tests (ALT, AST and bilirubin) and hospital outcomes 3. To evaluate the incidence of Remdisivir associated hepatotoxicity |
| **Proposed Target Population** |
| All patients with laboratory confirmed COVID-19 admitted to a hospital and for whom data is available in the ISARIC database |
| **Clinical Questions/Descriptive Analyses** |
| Serum bilirubin, ALT, and AST at baseline will be considered for analysis. For the purposes of this analysis, normal upper limits (ULN) for serum bilirubin, ALT and AST will be taken as 1 mg/dL, 40 U/L and 40 U/L respectively.  Each patient will be assigned a Liver Injury Classification score at Baseline (LIC-1) based on the 3 components of LFT on admission: stage I) Normal: all 3 components <= ULN; Stage II) Liver injury: any 1 component between 1-3x ULN; Stage III) Severe liver injury: any 1 component >= 3x ULN.  Patients who undergo a repeat LFT during hospital stay within 3-5 days from the initial LFT will be similarly assigned Liver Injury Classification during Hospitalization (LIC-2).  **Clinical questions include:**  1) What is the prevalence (overall and according to Liver injury Classification) of LFT (ALT/AST/Bilirubin) derangements in COVID-19 patients at presentation and during hospitalization? We will also evaluate if there is there an association between prior CLD status and LFT levels at admission.  2) What are the baseline characteristics of COVID-19 patients stratified by  i) Liver Injury Classification at Baseline (LIC-1):  a) Normal  b) Liver Injury  c) Severe Liver Injury  where baseline characteristics include  a) key demographic variables (age, sex, comorbidity profile, pregnancy)  b) clinical variables [presenting symptoms- a history of self-reported feverishness or measured fever of > 38° C, cough, dyspnoea (shortness of breath) , headache, altered consciousness / confusion, seizures, Abdominal pain, vomiting / nausea, diarrhoea), mean oxygen saturation at admission, time from symptom onset in days]  c) laboratory biomarkers on admission (ferritin, D-dimer, CRP, ESR, LDH, IL-6, Troponin, Creatine Kinase and procalcitonin)  d) clinical events at hospital admission (requirements of intensive care unit (ICU) admission, oxygen therapy, non-invasive ventilation (NIV), invasive ventilation, inotrope/vasopressor, renal replacement therapy)  3) What are the systemic complications and clinical outcomes in COVID-19 patients stratified by Liver Injury Classification at baseline (LIC-1) and during the hospital stay (LIC-  a) Normal  b) Liver Injury  c) Severe Liver Injury,  where  systemic complications include ARDS, shock, cardiac arrest, acute kidney injury, and bleeding,  AND  Clinical outcomes include:  a) Intensive care unit (ICU) admission, need for oxygen therapy, need for non-invasive ventilation (NIV), need for invasive ventilation, need for inotrope/vasopressor, need for ECMO and RRT  b) Duration of hospital and ICU stay, mortality  4) What is the incidence of Remdesivir hepatotoxicity (Grade 3/4 Hepatic adverse events defined as > 5x ULN of ALT/AST) in patients with normal and abnormal LFT at baseline? |
|  |
| **Planned Statistical Analyses, Methodology and Representation** |
| \| P value < .05 is to be deemed as statistically significant differences. All analyses will be two-tailed.  Statistical analysis will be performed using Stata or R or SAS. \| \| \| \| --- \| --- \| --- \| \| Clinical Question \| Planned statistical analysis \| Planned representation in manuscript \| \| 1) What is the prevalence (overall and according to Liver injury Classification) of LFT (ALT/AST/Bilirubin) derangements in COVID-19 patients at presentation and during hospitalization? \| Overall frequencies and percentages of patients having serum values of < ULN, 1-3X ULN and >3X ULN for ALT, AST and serum bilirubin at presentation and during hospitalization.  Overall frequencies and percentages of patients in each category of LIC-1 and LIC-2 (Normal, Liver Injury and Severe Liver Injury) \| Bar plots or summary tables for descriptive analysis \| \| 2) What are the baseline characteristics of COVID-19 patients stratified by Liver Injury Classification at Baseline (LIC-1) ? \| Comparisons across the stages of LIC-1 will be done using Kruskal-Wallis rank sum test. \| Summary tables \| \| 3) What are the systemic complications and clinical outcomes in COVID-19 patients stratified by Liver Injury Classification at Baseline (LIC-1) and during hospitalization (LIC-2)? \| Univariable and multivariable logistic regression analysis will be used to evaluate the association between LIC-1 levels, demographic, and clinical characteristics with outcomes and systemic complications.  Random effects for countries will be used to account for clustering. \| Tables presenting the Odds ratios and 95% CI of logistic regressions \| \| 4) What is the incidence of Remdesivir hepatotoxicity (Grade 3/4 Hepatic adverse events defined as > 5x ULN of ALT/AST) in patients with normal and abnormal LFT at baseline? \| Logistic regression will be used to evaluate the association between the administration of Remdesivir and hepatoxicity, while adjusting for demographics, clinical characteristics, comorbidities, and hepatic levels upon admission to hospital.  Random effects for countries will be used to account for clustering. \| Tables presenting the Odds ratios and 95% CI of logistic regressions \| |
| **Handling of Missing Data** |
| Preliminary analysis would be performed to ascertain a detailed overview of the extent of missingness in the data. This should enable the identification of variables which lack sufficient data to allow for any useful analysis to performed on them. Type of missingness shall be considered including whether data are not missing at random. Variables with greater than 30% missingness will be excluded from analysis. Where appropriate, imputation will be performed using Multiple Imputation by Chained Equations (MICE). |

# Other Information/Timelines

Circulation of proposal and receipt of feedback from partners: July 15^th^- Aug 15^th^ 2021

Analysis: Aug 15^th^ 2021- October 15^th^ 2021

Manuscript preparation and circulation for feedback: October 15^th^ – December 15^th^ 2021

Publication: January 2022

# References

1. Chen N, Zhou M, Dong X, Qu J, Gong F, Han Y, et al. Epidemiological and clinical characteristics of 99 cases of 2019 novel coronavirus pneumonia in Wuhan, China: a descriptive study. Lancet 2020;395:507–13.
2. Guan WJ, Ni ZY, Hu Y, Liang WH, Ou CQ, He JX, et al. Clinical Characteristics of Coronavirus Disease 2019 in China. N Engl J Med 2020;382:1708–20.
3. Shi H, Han X, Jiang N, et al. Radiological findings from 81 patients with COVID-19 pneumonia in Wuhan, China: a descriptive study. Lancet Infect Dis 2020;20(4):425-34.
4. Huang Y, Zhou H, Yang R, et al. Clinical characteristics of 36 non-survivors with COVID-19 in Wuhan, China. medRxiv;2020; in press. https://doi.org/10.1101/2020.02.27.20029009
5. Fu, Y. et al. Clinical features of COVID-19-infected patients with elevated liver biochemistries: a multicenter, retrospective study. *Hepatology* https://doi.org/10.1002/hep.31446 (2020).
6. Cai Q, Huang D, Ou P, et al. COVID-19 in a Designated Infectious Diseases Hospital Outside Hubei Province, China. Allergy 2020;20(4):425-34.
7. Anastasiou O, E, Korth J, Herbstreit F, Witzke O, Lange C, M: Mild versus Severe Liver Injury in SARS-CoV-2 Infection. Dig Dis 2020. doi: 10.1159/000510758
8. Wang Q , Zhao H , Liu LG , Wang YB , Zhang T , Li MH , et al. Pattern of liver injury in adult patients with COVID-19: a retrospective analysis of 105 patients. Military Medical Research 2020;7(1):28. https://doi.org/10.1186/s40779-020-00256-6

1. Either chair and/or co-chair are based in an institution in an LMIC. If you would like to be connected with an eligible co-chair please let us know at ncov@isaric.org. [↑](#footnote-ref-1)
