# Supplementary material for: Liver injury in hospitalized patients with COVID-19: An International observational cohort study
Source: PLoS One. 2023 Sep 13;18(9):e0277859. doi: 10.1371/journal.pone.0277859 (PMC10499210; doi:10.1371/journal.pone.0277859)
Supplement: Attachment — Submitted filename: Response to Editorial and Reviewer comments.docx [file pone.0277859.s008.docx]

**Response to Editorial and Reviewer comments**

We thank the Editor and the Reviewers for the feedback. The comments and suggestions have helped us improve our manuscript and we are grateful for this. We provide a point-by-point response to each of the comments. Editor and reviewer comments are italicized, and our responses are listed below each comment.

*1. Please ensure that your manuscript meets PLOS ONE's style requirements, including those for file naming. The PLOS ONE style templates can be found at*

[*https://journals.plos.org/plosone/s/file?id=wjVg/PLOSOne_formatting_sample_main_body.pdf*](about:blank) *and*

[*https://journals.plos.org/plosone/s/file?id=ba62/PLOSOne_formatting_sample_title_authors_affiliations.pdf*](about:blank)*.*

**Response**: Thank you. We have checked and revised to meet these requirements.

*2. Please provide additional details regarding participant consent. In the ethics statement in the Methods and online submission information, please ensure that you have specified what type you obtained (for instance, written or verbal, and if verbal, how it was documented and witnessed). If your study included minors, state whether you obtained consent from parents or guardians. If the need for consent was waived by the ethics committee, please include this information.*

*Once you have amended this/these statement(s) in the Methods section of the manuscript, please add the same text to the “Ethics Statement” field of the submission form (via “Edit Submission”).*

*For additional information about PLOS ONE ethical requirements for human subjects research, please refer to* [*http://journals.plos.org/plosone/s/submission-guidelines#loc-human-subjects-research*](about:blank#loc-human-subjects-research)*.*

**Response**: Our manuscript is a **secondary analysis** of data available in the International Severe Acute Respiratory and emerging Infection Consortium(ISARIC) COVID-19 dataset. As such, direct patient-level consent is not applicable for this analysis, but was obtained where applicable and required for primary data collection and reuse. At the onset of the pandemic, ISARIC in collaboration with the World Health Organization developed a clinical characterization protocol and a standardized case report form (CRF) for harmonized collection of data across hospitals and countries. The CRF was made available as an open-access resource and a central REDCAP database was hosted by ISARIC. Sites across the world had the option of contributing data to this central database after obtaining appropriate local ethics approvals and patient-level consent (if deemed necessary as per site and country requirements).

The ISARIC-WHO Clinical Characterisation Protocol was approved by the World Health Organization Ethics Review Committee (RPC571 and RPC572 on 25 April 2013). Institutional approval was additionally obtained by participating sites including the South Central Oxford C Research Ethics Committee in England (Ref 13/SC/0149) and the Scotland A Research Ethics Committee (Ref 20/SS/0028) for the United Kingdom, representing the majority of the data. Other institutional and national approvals were obtained by participating sites as per local requirements. Regionally appropriate decisions regarding a waiver or requirement of patient consent were made by each committee and implemented at the sites.

*3. Thank you for stating the following in the Acknowledgments/ Funding Section of your manuscript:*

*“This work was made possible by the UK Foreign, Commonwealth and Development Office*

*and Wellcome [215091/Z/18/Z, 222410/Z/21/Z, 225288/Z/22/Z and 220757/Z/20/Z]; the Bill*

*&amp; Melinda Gates Foundation [OPP1209135]; the philanthropic support of the donors to the*

*University of Oxford’s COVID-19 Research Response Fund (0009109); CIHR Coronavirus*

*Rapid Research Funding Opportunity OV2170359 and the coordination in Canada by*

*Sunnybrook Research Institute; endorsement of the Irish Critical Care- Clinical Trials Group,*

*co-ordination in Ireland by the Irish Critical Care- Clinical Trials Network at University*

*College Dublin and funding by the Health Research Board of Ireland [CTN-2014-12]; the*

*COVID clinical management team, AIIMS, Rishikesh, India; Cambridge NIHR Biomedical*

*Research Centre; the dedication and hard work of the Groote Schuur Hospital Covid ICU*

*Team and supported by the Groote Schuur nursing and University of Cape Town registrar*

*bodies coordinated by the Division of Critical Care at the University of Cape Town; the*

*Liverpool School of Tropical Medicine and the University of Oxford; the dedication and hard*

*work of the Norwegian SARS-CoV-2 study team and the Research Council of Norway grant*

*no 312780, and a philanthropic donation from Vivaldi Invest A/S owned by Jon Stephenson*

*von Tetzchner; Imperial NIHR Biomedical Research Centre; the Comprehensive Local*

*Research Networks (CLRNs) of which PJMO is an NIHR Senior Investigator*

*(NIHR201385); Innovative Medicines Initiative Joint Undertaking under Grant Agreement*

*No. 115523 COMBACTE, resources of which are composed of financial contribution from*

*the European Union’s Seventh Framework Programme (FP7/2007- 2013) and EFPIA*

*companies, in-kind contribution; Stiftungsfonds zur Förderung der Bekämpfung der*

*Tuberkulose und anderer Lungenkrankheiten of the City of Vienna, Project Number:*

*APCOV22BGM; Italian Ministry of Health “Fondi Ricerca corrente–L1P6” to IRCCS*

*Ospedale Sacro Cuore–Don Calabria; Australian Department of Health grant (3273191);*

*Gender Equity Strategic Fund at University of Queensland, Artificial Intelligence for*

*Pandemics (A14PAN) at University of Queensland, the Australian Research Council Centre*

*of Excellence for Engineered Quantum Systems (EQUS, CE170100009), the Prince Charles*

*Hospital Foundation, Australia; Brazil, National Council for Scientific and Technological*

*Development Scholarship number 303953/2018- 7; the Firland Foundation, Shoreline,*

*Washington, USA;  the French COVID cohort (NCT04262921) is sponsored by INSERM*

*and is funded by the REACTing (REsearch &amp; ACtion emergING infectious diseases)*

*consortium and by a grant of the French Ministry of Health (PHRC n°20-0424); a grant from*

*foundation Bevordering Onderzoek Franciscus; the South Eastern Norway Health Authority*

*and the Research Council of Norway; Institute for Clinical Research (ICR), National*

*Institutes of Health (NIH) supported by the Ministry of Health Malaysia; preparedness work*

*conducted by the Short Period Incidence Study of Severe Acute Respiratory Infection.*

*This work uses data provided by patients and collected by the NHS as part of their care and*

*support #DataSavesLives. The data used for this research were obtained from ISARIC4C. We*

*are extremely grateful to the 2648 frontline NHS clinical and research staff and volunteer*

*medical students who collected these data in challenging circumstances; and the generosity of*

*the patients and their families for their individual contributions in these difficult times. The*

*COVID-19 Clinical Information Network (CO-CIN) data was collated by ISARIC4C*

*Investigators. Data and Material provision was supported by grants from: the National*

*Institute for Health Research (NIHR; award CO-CIN-01), the Medical Research Council*

*(MRC; grant MC_PC_19059), and by the NIHR Health Protection Research Unit (HPRU) in*

*Emerging and Zoonotic Infections at University of Liverpool in partnership with Public*

*Health England (PHE), (award 200907), NIHR HPRU in Respiratory Infections at Imperial*

*College London with PHE (award 200927), Liverpool Experimental Cancer Medicine Centre*

*(grant C18616/A25153), NIHR Biomedical Research Centre at Imperial College London*

*(award ISBRC-1215-20013), and NIHR Clinical Research Network providing infrastructure*

*support. We also acknowledge the support of Jeremy J Farrar and Nahoko Shindo.”*

*We note that you have provided funding information that is not currently declared in your Funding Statement. However, funding information should not appear in the Acknowledgments section or other areas of your manuscript. We will only publish funding information present in the Funding Statement section of the online submission form.*

*Please remove any funding-related text from the manuscript and let us know how you would like to update your Funding Statement. Currently, your Funding Statement reads as follows:*

*“Recipient: Funding received by the International Severe Acute Respiratory and emerging Infection Consortium (ISARIC).*

*This work was made possible by the UK Foreign, Commonwealth and Development Office and Wellcome [215091/Z/18/Z, 222410/Z/21/Z, 225288/Z/22/Z and 220757/Z/20/Z]; the Bill*

*& Melinda Gates Foundation [OPP1209135];*

*URL:*

[*https://wellcome.org/*](about:blank)

[*https://www.gov.uk/government/organisations/foreign-commonwealth-development-office*](about:blank)

*The funders had no role in study design, data collection and analysis, decision to publish, or preparation of the manuscript”*

*Please include your amended statements within your cover letter; we will change the online submission form on your behalf.*

**Response**: Thank you. We have now deleted references to funding in the acknowledgements section. Our revised funding statement is as below:

“This work was made possible by the UK Foreign, Commonwealth and Development Office and Wellcome [215091/Z/18/Z, 222410/Z/21/Z, 225288/Z/22/Z]; and the Bill & Melinda Gates Foundation [OPP1209135]. The funders had no role in the design, analysis, manuscript preparation or decision to submit for publication. “

*4. In your Data Availability statement, you have not specified where the minimal data set underlying the results described in your manuscript can be found. PLOS defines a study's minimal data set as the underlying data used to reach the conclusions drawn in the manuscript and any additional data required to replicate the reported study findings in their entirety. All PLOS journals require that the minimal data set be made fully available. For more information about our data policy, please see* [*http://journals.plos.org/plosone/s/data-availability*](about:blank)*.*

*Upon re-submitting your revised manuscript, please upload your study’s minimal underlying data set as either Supporting Information files or to a stable, public repository and include the relevant URLs, DOIs, or accession numbers within your revised cover letter. For a list of acceptable repositories, please see* [*http://journals.plos.org/plosone/s/data-availability#loc-recommended-repositories*](about:blank#loc-recommended-repositories)*. Any potentially identifying patient information must be fully anonymized.*

*Important: If there are ethical or legal restrictions to sharing your data publicly, please explain these restrictions in detail. Please see our guidelines for more information on what we consider unacceptable restrictions to publicly sharing data:* [*http://journals.plos.org/plosone/s/data-availability#loc-unacceptable-data-access-restrictions*](about:blank#loc-unacceptable-data-access-restrictions)*. Note that it is not acceptable for the authors to be the sole named individuals responsible for ensuring data access.*

*We will update your Data Availability statement to reflect the information you provide in your cover letter.*

**Response:** Thank you. Apologies for the lack of clarity. The minimal dataset underlying the study results are already available within the manuscript.

For any additional data, we have expanded on the process for seeking access under the ‘Data Availability Statement’ and is reproduced below:

“The minimal dataset underlying the results are available within the manuscript and supplementary files. Any additional data that relate to this analysis are highly detailed clinical data on individuals hospitalised with COVID-19. Due to the sensitive nature of these data and the associated privacy concerns, they are available via a governed data access mechanism following review of a data access committee. Data can be requested via the IDDO COVID-19 Data Sharing Platform (http://www.iddo.org/covid-19). The Data Access Application, Terms of Access and details of the Data Access Committee are available on the website. Briefly, the requirements for access are a request from a qualified researcher working with a legal entity who have a health and/or research remit; a scientifically valid reason for data access which adheres to appropriate ethical principles. The full terms are at https://www.iddo.org/document/covid-19-data-access-guidelines. A small subset of sites who contributed data to this analysis have not agreed to pooled data sharing as above. In the case of requiring access to these data, please contact the corresponding author in the first instance who will look to facilitate access.”

*5. We note that you have indicated that data from this study are available upon request. PLOS only allows data to be available upon request if there are legal or ethical restrictions on sharing data publicly. For more information on unacceptable data access restrictions, please see* [*http://journals.plos.org/plosone/s/data-availability#loc-unacceptable-data-access-restrictions*](about:blank#loc-unacceptable-data-access-restrictions)*.*

*In your revised cover letter, please address the following prompts:*

*a) If there are ethical or legal restrictions on sharing a de-identified data set, please explain them in detail (e.g., data contain potentially sensitive information, data are owned by a third-party organization, etc.) and who has imposed them (e.g., an ethics committee). Please also provide contact information for a data access committee, ethics committee, or other institutional body to which data requests may be sent.*

*b) If there are no restrictions, please upload the minimal anonymized data set necessary to replicate your study findings as either Supporting Information files or to a stable, public repository and provide us with the relevant URLs, DOIs, or accession numbers. For a list of acceptable repositories, please see* [*http://journals.plos.org/plosone/s/data-availability#loc-recommended-repositories*](about:blank#loc-recommended-repositories)*.*

*We will update your Data Availability statement on your behalf to reflect the information you provide.*

**Response:** Thank you. We have expanded on this in response to this previous question.

*6. "One of the noted authors is a group or consortium [ISARIC Clinical Characterisation Group]. In addition to naming the author group, please list the individual authors and affiliations within this group in the acknowledgments section of your manuscript. Please also indicate clearly a lead author for this group along with a contact email address."*

**Response:** Thank you. The lead author for the group is Laura Merson (Email address: laura.merson@ndm.ox.ac.uk). The full list of authors with affiliations is attached as a *csv* file with details (supplementary material). Providing the affiliations for each author in the acknowledgements would lead to a substantial increase in the number of pages (over 20 pages in a word document). We have taken this approach of listing only the author names in a previous PLoS publication without any objections (https://journals.plos.org/plosmedicine/article?id=10.1371/journal.pmed.1003969#abstract2). Please let us know if this is okay.

*7. Please review your reference list to ensure that it is complete and correct. If you have cited papers that have been retracted, please include the rationale for doing so in the manuscript text, or remove these references and replace them with relevant current references. Any changes to the reference list should be mentioned in the rebuttal letter that accompanies your revised manuscript. If you need to cite a retracted article, indicate the article’s retracted status in the References list and also include a citation and full reference for the retraction notice.*

**Response:** Thank you. We have checked and the reference list is complete and correct.

*Reviewer 1:*

*Well done! Very interesting findings that can contribute to more effective way in managing COVID-19 patients. There are some formatting and standard English errors that need to be reviewed. Some of the information can be misleading and confusing. The conclusion needs to be more impactful.*

Response: Thank you for the encouraging and positive feedback. We have reviewed the manuscript in detail and corrected any formatting/English errors.

We have edited the conclusions. However, given the nature of the analysis (retrospective secondary analysis of observational information from a dataset) and the limitations of our work, we have framed our conclusions cautiously.

The revised conclusion is as below:

“Liver enyzme abnormalities are common among COVID-19 patients and associated with worse outcomes. Multiple mechanisms may explain the extent and severity of liver injury in COVID-19. Future research should focus on understanding these mechanisms, the impact of changes over time, and whether antivirals improve or worsen liver injury.”
